# Assessing Zika virus replication and the development of Zika-specific antibodies after a mid-gestation viral challenge in guinea pigs

**DOI:** 10.1371/journal.pone.0187720

**Published:** 2017-11-03

**Authors:** Craig J. Bierle, Claudia Fernández-Alarcón, Nelmary Hernandez-Alvarado, Jason C. Zabeli, Bradley C. Janus, Dira S. Putri, Mark R. Schleiss

**Affiliations:** Division of Pediatric Infectious Disease and Immunology, Department of Pediatrics, University of Minnesota, Minneapolis, Minnesota, United States of America; CEA, FRANCE

## Abstract

Primary Zika virus (ZIKV) infections that occur during pregnancy can cause spontaneous abortion and profoundly disrupt fetal development. While the full range of developmental abnormalities associated with congenital Zika syndrome is not yet known, severe cases of the syndrome can present with microcephaly, extensive neurologic and ocular damage, and pronounced joint malformations. Animal models that accurately recapitulate congenital Zika syndrome are urgently needed for vaccine development and for the study of ZIKV pathogenesis. As guinea pigs have successfully been used to model transplacental infections by cytomegalovirus, syphilis, and *Listeria monocytogenes*, we sought to test whether ZIKV could productively infect guinea pigs and whether viral transmission with attendant fetal pathology would occur after a mid-gestation viral challenge. We found that guinea pig cells supported ZIKV replication *in vitro*. Experimental infection of non-pregnant animals did not result in overt disease but low-level, detectable viremia was observed. When pregnant guinea pigs were challenged with ZIKV at between 18 and 21 days gestational age, ZIKV was not detected in maternal or pup blood, plasma, or tissues and no significant differences in maternal weight gain or pup size were observed following challenge. Nonetheless, a robust antibody response against ZIKV was detected in both the pups and dams. These results suggest that, while guinea pigs can model aspects of the immune response to ZIKV infection during pregnancy, naturally circulating ZIKV strains are not pathogenic during the pregnancy of immunocompetent guinea pigs and do not interfere with normal pup development.

## Introduction

Zika virus (ZIKV) is an emerging, mosquito-borne flavivirus that can be transmitted transplacentally and disrupt the normal development of human fetuses. Prior to the recent outbreaks in the Pacific and Americas, ZIKV infection was believed to only cause a mild febrile illness characterized by fever, rash, arthralgia, myalgia, headache, and conjunctivitis [1]. ZIKV associated Guillain-Barré syndrome was initially observed during the 2013 outbreak in French Polynesia, complicating an estimated 0.024% of ZIKV infections [2, 3]. An unusual spike in the incidence of microcephaly in Brazil was first observed in 2015 and led to the discovery of *in utero* ZIKV transmission and congenital Zika syndrome [4–6]. ZIKV infection during pregnancy is associated with prolonged viremia and virus has been detected in the serum of pregnant women up to 53 days post-exposure [7]. Birth defects have been observed in 6% of completed pregnancies complicated by maternal ZIKV infection, and maternal infection during the first trimester of pregnancy appears to confer the greatest risk of injury to the fetus [8].

ZIKV pathogenesis *in vivo* and the mechanisms responsible for viral transmission across the placenta are poorly understood. To address this deficiency, multiple animal models of ZIKV infection have been developed. Experimental ZIKV challenges in macaques have revealed that infection in nonhuman primates shares many features with ZIKV disease in humans. Infections in nongravid macaques cause short-term viremia that generally resolves by ten days post-infection [9–11]. Infections in pregnant macaques result in a prolonged period of viremia and ZIKV has been detected in the blood of gravid animals up to seventy days post-infection [10, 12]. Viral RNA and histopathologic evidence of viral infection was observed in a variety of fetal tissues, including the brain, eye, and placenta, after an experimental ZIKV challenge during rhesus macaque pregnancy [12]. Intrauterine transmission of ZIKV was also observed in a separate study where a pigtail macaque was infected with ZIKV mid-gestation [13]. In this study, clinical disease was not detected in the ZIKV challenged dam, but ZIKV was detected in multiple maternal tissues and the fetus’s brain had multiple injuries, including white matter deficiency, gliosis, and axonal damage [13].

The development of small animal models of congenital ZIKV syndrome has been impeded by the non-permissivity of rodents to infection. Naturally occurring ZIKV strains are not virulent in wild-type (WT) C57BL/6, BALB/c, or CD-1 mice, a phenotype that may be attributed to an inability of the virus to evade murine STAT2 [14, 15]. Disruption of normal interferon α/β or STAT2 signaling significantly increases the pathogenicity of ZIKV in mice and facilitates transplacental transmission of the virus [16, 17]. A murine model of congenital ZIKV syndrome in immunocompetent mice remains elusive, although transplacental ZIKV transmission and fetal intrauterine growth restriction were observed in SJL mice when an exceptionally high dose of virus (4X10^10^ PFU per animal) was administered intravenously to pregnant dams [18].

Guinea pigs are broadly used as an experimental model for congenitally and sexually transmitted diseases. Unlike laboratory mice and rats, guinea pigs and humans both have hemomonochorial placentas and share a homologous mechanism of trophoblast invasion [19, 20]. Whereas murid rodents have short gestational periods and birth altricial young, guinea pig pregnancy is long (65–70 d), the pups are born precocial, and guinea pigs and humans share many features of *in utero* neural development. Experimental models of congenital cytomegalovirus, listeriosis, syphilis, and toxoplasmosis in guinea pigs have been developed [21–24].

The utility of guinea pigs for studying ZIKV pathogenesis *in utero* remains unclear. In early studies performed shortly after ZIKV was discovered, two animals inoculated intracerebrally with low-passage, Ugandan MR 766 ZIKV died 6 days post challenge, but no information was available regarding the pathology of the presumed infection [25]. This experiment could not be repeated due to the limited availability of low-passage virus and guinea pigs challenged with MR 766 that had been extensively passaged in mice showed no signs of infection [25]. Because MR 766 has been passaged intracerebrally in mice, the virus has likely adapted to this non-natural host and route of infection. More recently, juvenile guinea pigs challenged with low-passage ZIKV isolated from Puerto Rico in 2015 (PRVABC 59) demonstrated clinical signs of infection, including fever, lethargy, hunched back, ruffled fur, and a decrease in mobility [26]. Low-level, acute viremia was detected by qRT-PCR and plaque assay in infected animals 2–3 days post-challenge and virus was recovered from the brains of challenged guinea pigs [26]. The effect of ZIKV infection during guinea pig pregnancy has not been previously described.

To determine whether ZIKV can cause fetal infection and disease in guinea pigs, we evaluated the pathogenicity of ZIKV in time-mated animals. Consistent with previously published work, low-level viremia and antigenemia occurred when non-pregnant guinea pigs were challenged with ZIKV isolated from the 2013 outbreak in French Polynesia (H/PF/2013) [26, 27]. However, neither obvious signs of clinical disease nor viremia were observed in guinea pig dams challenged during pregnancy, nor was virus recovered from placental or pup tissue. A robust antibody response against ZIKV did develop in the infected dams and anti-ZIKV antibodies were recovered from their pups. Together, these results suggest that naturally occurring ZIKV strains are immunologically well-controlled by pregnant guinea pigs and that either immune suppression or host-adaptation of ZIKV strains will be necessary if a guinea pig model of congenital Zika syndrome is to be developed.

## Results

### Guinea pig cells are permissive to Zika virus infection

**Fig 1:**
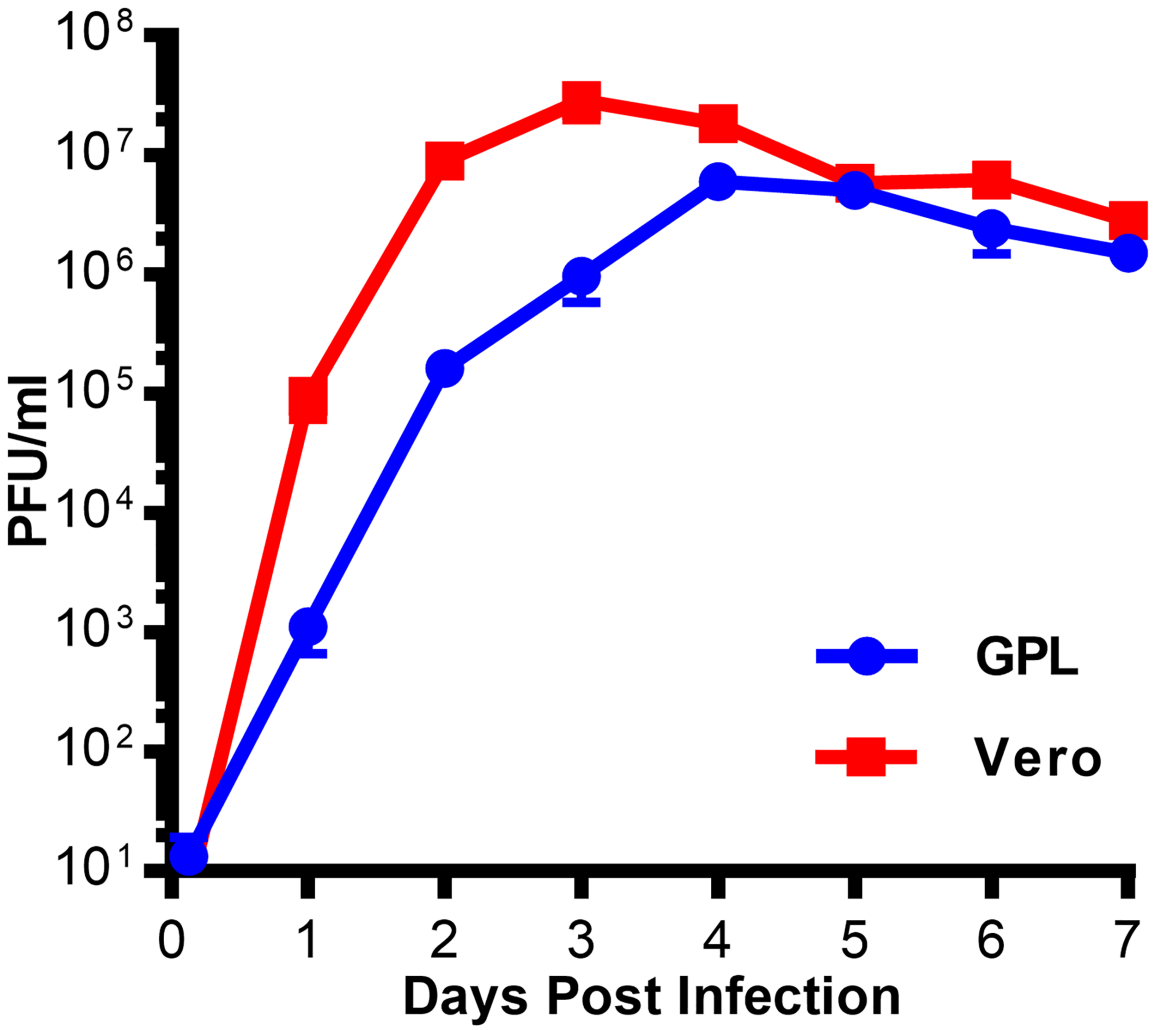
ZIKV infection of guinea pig cells *in vitro*. GPL and Vero cells were infected at a low MOI with ZIKV. Cell culture supernatant was collected daily and titered on Vero cells. The mean titer from three replicate infections is shown, and the bars represent the standard error for each set of replicates.

Previous studies have reported that ZIKV could cause clinical disease in guinea pigs either following an intracerebral inoculation using an African lineage virus or after subcutaneous challenge with a recent Puerto Rican isolate [25, 26]. To determine whether ZIKV can replicate in guinea pig cells *in vitro*, guinea pig lung fibroblasts (JH4) and Vero cells were infected at a low multiplicity of infection (MOI). Cell culture supernatant was collected from these cells daily and titered on Vero cells in a multi-step growth curve analysis (Fig 1). Both JH4 and Vero cells supported ZIKV replication. While ZIKV replication and maximal titers were delayed and reduced in JH4 cells compared to Vero cells, this experiment demonstrated that guinea pig cells were permissive to ZIKV and that there was not an intrinsic blockade to infection.

### Zika virus causes viremia and antigenemia in non-pregnant guinea pigs

**Fig 2:**
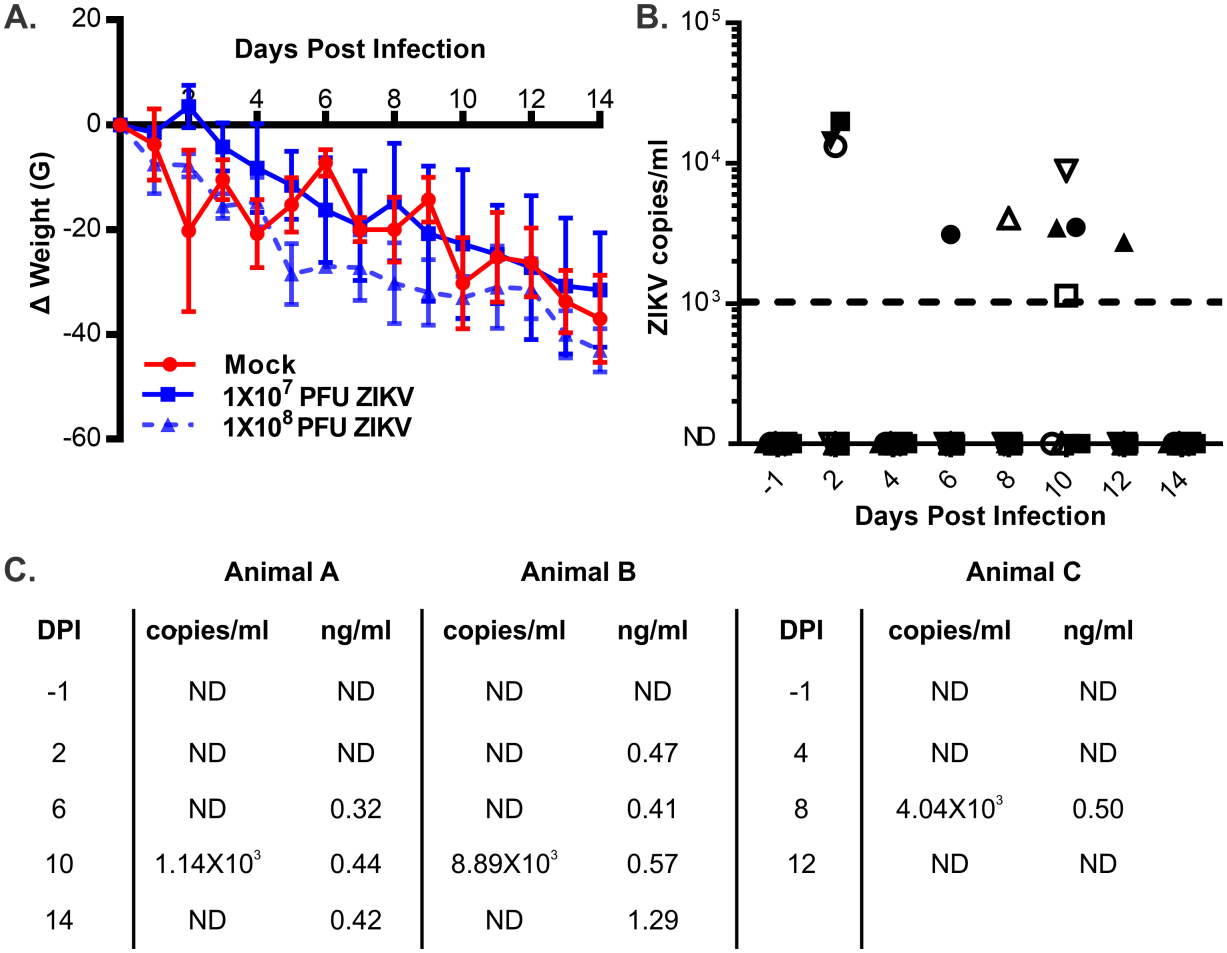
ZIKV infection of non-pregnant guinea pigs. Groups of guinea pigs (N = 4) were infected with 1x10^7^ or 1X10^8^ PFU of ZIKV or sham injected. **(A)** The weight change of experimentally challenged guinea pigs over time was recorded. The mean weight change in each group of challenged animals and the standard error at each time point is plotted. **(B)** Viremia in whole blood from challenged guinea pigs was quantified using qRT-PCR. Solid symbols indicate animals challenged with 1X10^7^ PFU; open symbols indicate animals challenged with 1X10^8^ PFU. **(C)** Select plasma samples from guinea pigs challenged with 1X10^8^ PFU of ZIKV were examined using a ZIKV NS1 ELISA. The number of ZIKV genome copies/ml and the magnitude of antigenemia, as assessed in ng/ml NS1 in blood or plasma from each animal at the indicated time point post-infection are shown. ND: not detectable.

Having established that ZIKV replicates in guinea pig cells, we next sought to evaluate whether the virus productively infects guinea pigs. Previous research into the transmission of West Nile and dengue viruses has estimated that between 1X10^4^ and 1X10^7^ plaque forming units (PFUs) of flavivirus are delivered by a mosquito during a single blood feeding [28, 29]. The saliva of a single ZIKV-infected *Aedes aegypti* mosquito can contain up to 1X10^8^ copies of the viral genome [30]. During experimental ZIKV infections in macaques, productive, acute viral infections were established after challenge with 1X10^3^ to 1X10^6^ PFU of virus [10]. As ZIKV replicates poorly and transplacental transmission has only been reported after challenge with extremely high doses of virus in wild type mice, we elected to challenge guinea pigs with either 1X10^7^ or 1X10^8^ plaque forming units of ZIKV [14, 18]. Groups of four 6-month old guinea pigs (two male, two female) were challenged with either dose of ZIKV administered by subcutaneous injection into the dorsal neck or sham injected with PBS. The guinea pigs were followed for 14 days post-challenge. No significant differences in weight change over time were observed among the three groups of animals (Fig 2a). All of the guinea pigs in this study lost weight, which was likely attributable to stress associated with relocation from conventional to individually ventilated housing. ZIKV-specific qRT-PCR detected viral RNA in the whole blood of all eight ZIKV challenged animals (Fig 2b). The timing of viremia varied between individual animals, and most guinea pigs only had a single ZIKV positive blood sample.

Significant levels of the viral protein NS1 accumulate in the serum of humans and mice infected with dengue virus [31–33]. This led us to test whether NS1 antigenemia also occurred during ZIKV infection in guinea pigs. A subset of plasma samples from guinea pigs challenged with 1X10^8^ PFU of ZIKV were subjected to an ELISA to quantify circulating levels of ZIKV NS1. Three animals had low but detectable levels of NS1 in their plasma in addition to quantifiable viremia (Fig 2c). Together, these results support recent observations that ZIKV establishes acute, low-level infections in guinea pigs [26].

### Zika virus is not pathogenic during guinea pig pregnancy

**Fig 3:**
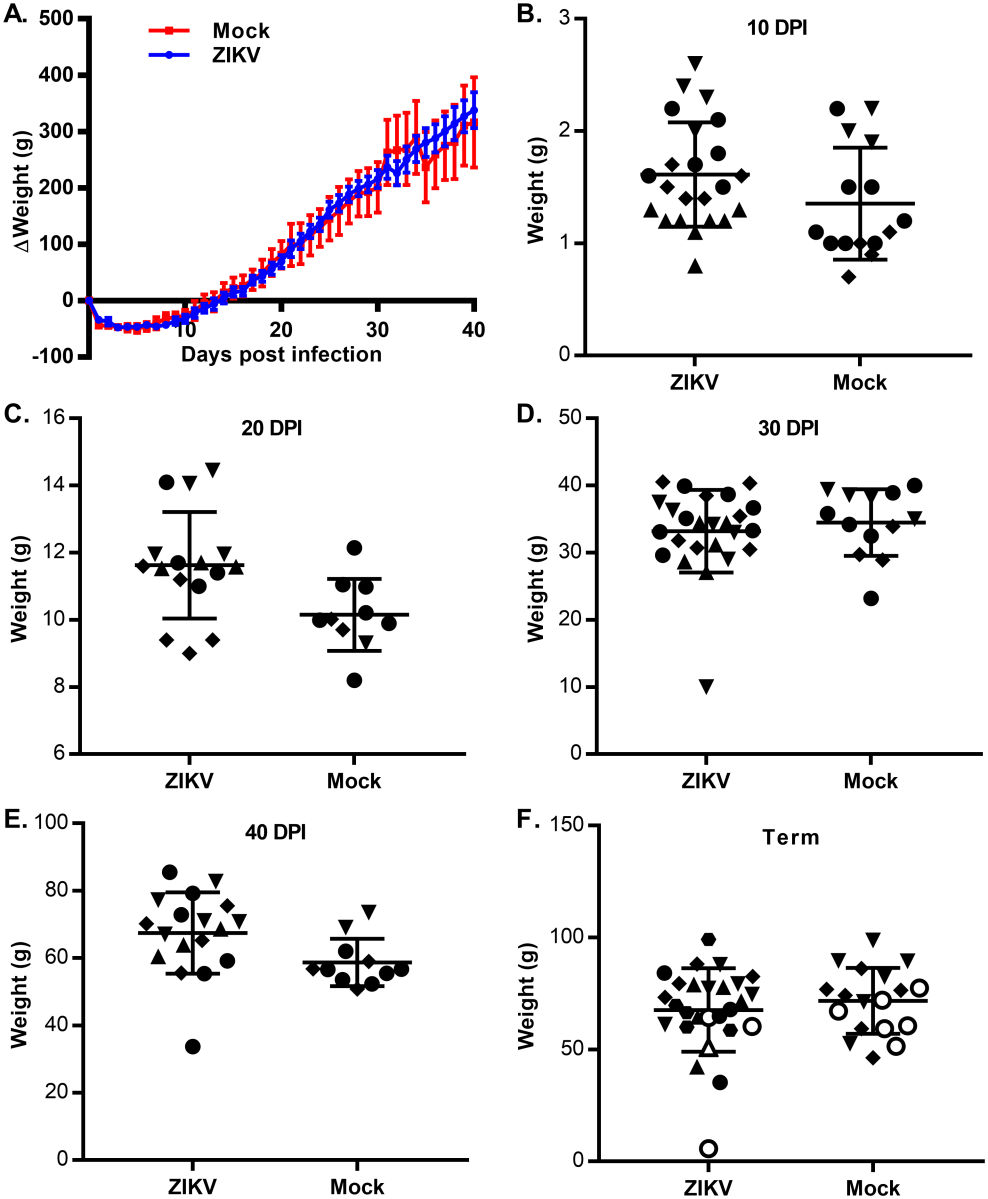
ZIKV infection of pregnant guinea pigs. Pregnant guinea pigs were infected with 1X10^7^ PFU of ZIKV (N = 21) or sham injected (N = 15) at GA 18–21. **(A)** Guinea pigs were weighed daily until a pre-determined experimental endpoint and the mean weight change for each group was plotted along with the standard error. Animals were euthanized at 10, 20, 30 or 40 days post-infection (N = 4 infected, 3 uninfected per group) or euthanized on the day of delivery (N = 5 infected, 3 uninfected per group). **(B-F)** Pups were weighed at the indicated endpoint post-infection. Symbol shape represents individual litters; open symbols indicate pups born dead. The bars indicate the standard error within each group.

To determine whether ZIKV is infectious in pregnant guinea pigs, guinea pigs were time-mated by breeding during postpartum estrus. Animals with plasma progesterone levels greater than 15 ng/ml were transferred to BSL2 housing and challenged with 1X10^7^ PFU of ZIKV or sham-injected with PBS subcutaneously at 18 to 21 days gestational age. The guinea pigs were challenged early in pregnancy because birth defects, including brain abnormalities, have been observed most frequently in humans when ZIKV infection occurs during the first trimester [8]. The guinea pig brain is largely undifferentiated at 18 days gestational age and rapidly differentiates in the following 14 day period [34]. Dams were weighed daily and whole blood and/or plasma were collected at regular intervals to assess the presence of viremia. Across multiple experiments that compared a total of 21 challenged dams with 15 sham-injected animals, no difference in the weight change over time was observed between ZIKV challenged and sham-injected dams (Fig 3a). While we observed transient viremia and NS1-antigenemia in ZIKV challenged non-gravid animals (Fig 2b and 2c), none of the blood or plasma samples from pregnant animals that were tested contained detectable ZIKV RNA or NS1 antigen.

Given the long (~65 day) gestation of guinea pigs, we anticipated that congenital ZIKV infection early in guinea pig pregnancy might resolve prior to birth. Therefore, in addition to comparing full-term pups born from ZIKV and mock-challenged animals, pregnant dams were euthanized at 10, 20, 30, and 40 days post-challenge. While the viability of pups could not be assessed when the animals were sacrificed preterm, placental tissues and amniotic fluid were collected from each pup that was euthanized at a preterm endpoint. While ZIKV challenge in pregnancy has been observed to cause *in utero* growth restriction in mouse models, pups from ZIKV challenged guinea pigs (N = 107) were not smaller than their mock challenged counterparts (N = 67) (Fig 3b–3f) [17, 18]. No ZIKV RNA was detected in challenged pup or placental tissues irrespective of the experimental endpoint. One of the three uninfected control animals spontaneously delivered a litter of dead pups one week before her expected due date. 4 of 27 pups from ZIKV challenged dams were born dead or partially resorbed, which represented a somewhat higher rate of fetal mortality than has been reported in Hartley guinea pigs [35]. However, the small group sizes and the lack of available in-house historic data on the rate of fetal mortality in guinea pigs bred during post-gestational estrus makes it impossible to conclude whether ZIKV infection contributed to *in utero* fetal demise. Together, these results suggest that pregnant guinea pigs successfully control ZIKV infection and that the virus did not infect either the placenta or guinea pig pups.

### Maternal antibodies to Zika virus are produced and transmitted transplacentally

**Fig 4:**
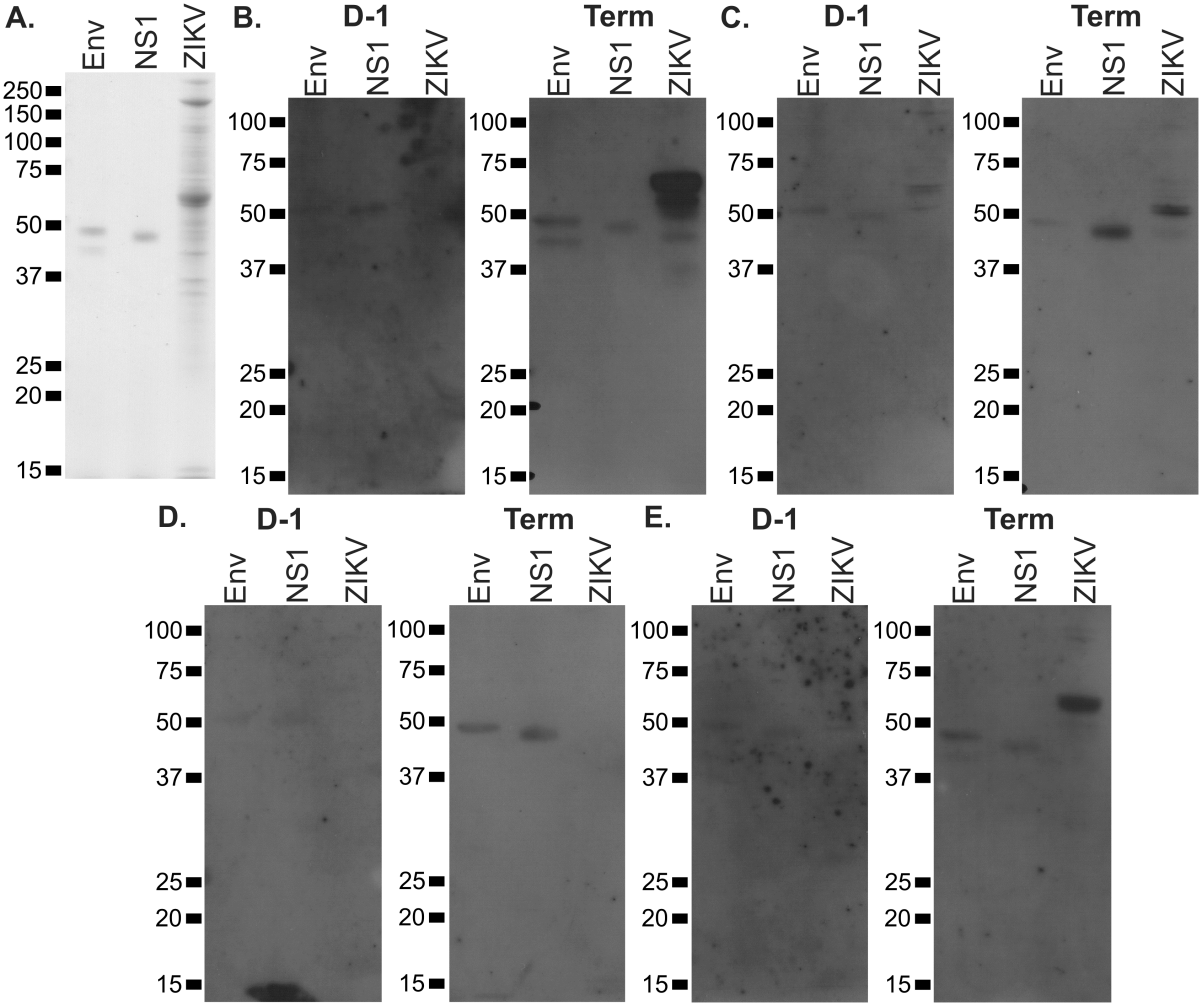
Anti-ZIKV antibody production by infected guinea pigs. To assess whether an antibody response to ZIKV proteins is elicited in challenged guinea pigs, purified ZIKV Env and NS1 and semi-purified viral particles were resolved by SDS-PAGE, transferred to membranes, and immunoblotted. **(A)** Coomassie stained gel showing the sizes of input protein. **(B-E)** Proteins were transferred to membranes and immunoblotted using plasma collected from four ZIKV challenged pregnant guinea pigs either the day before viral challenge (D-1) or the day of delivery (Term).

While the production of ZIKV RNA or antigen was not detected directly after experimental viral challenge during pregnancy, we evaluated the antibody response of pregnant guinea pigs to ZIKV. Recombinant ZIKV proteins (Env and NS1) and protein from partially-purified ZIKV particles was resolved by SDS-PAGE and immunoblotted using plasma from a subset of the guinea pigs that were challenged with ZIKV and allowed to deliver (~45d post challenge). Plasma collected after ZIKV challenge contained IgG antibodies that were immunoreactive to the recombinant viral proteins and viral inoculum (Fig 4).

**Fig 5:**
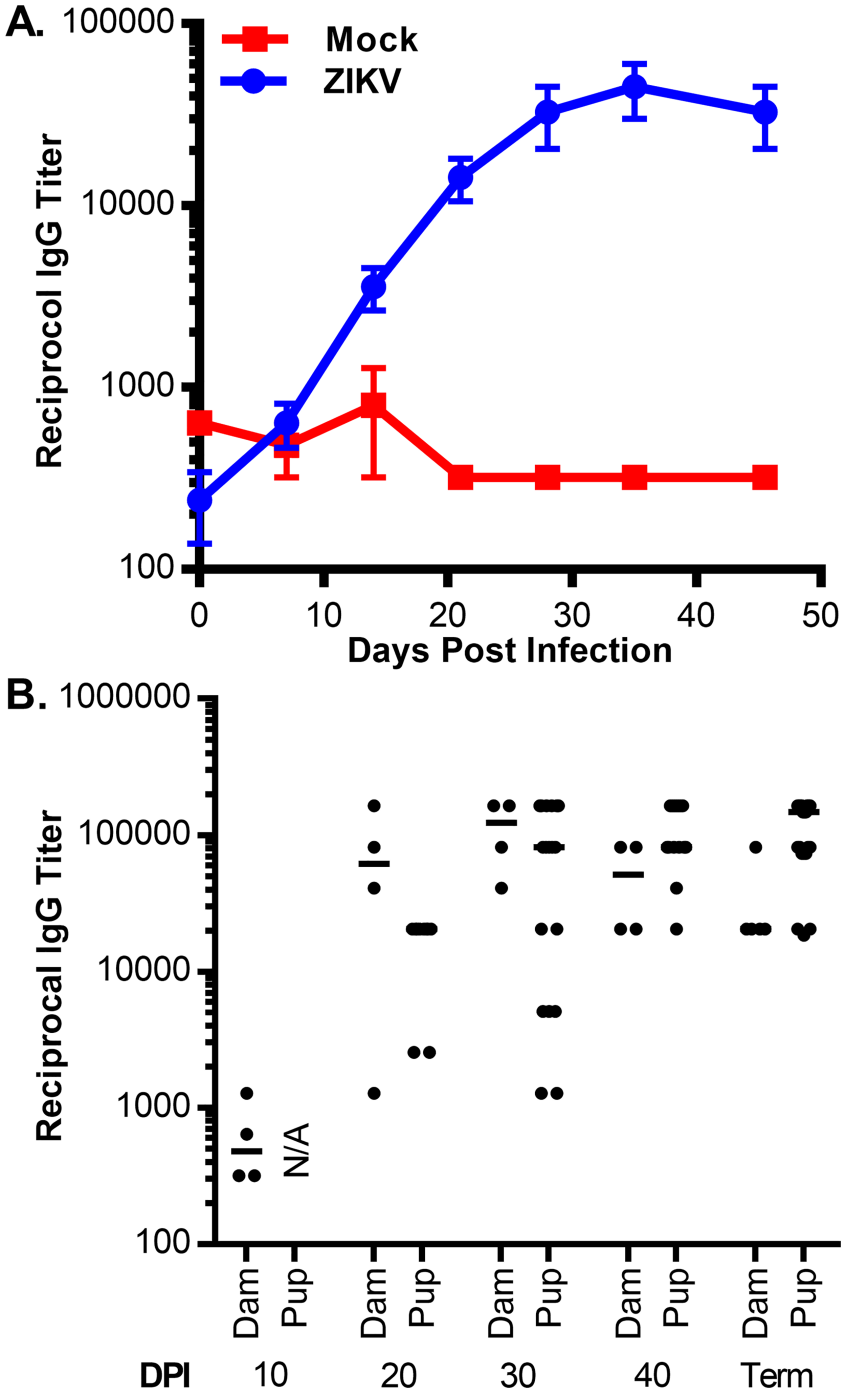
Evolution of the anti-NS1 response in pregnant dams. The IgG response to ZIKV NS1 of pregnant guinea pigs and their pups was measured by ELISA. **(A)** The mean anti-NS1 titer of pregnant guinea pigs challenged with ZIKV (N = 5) or sham injected (N = 2) was measured over time and plotted; bars indicate the standard error for each time point. **(B)** The anti-NS1 titers of pregnant dams and pups were measured at each experimental endpoint. The bars indicate the mean antibody titer at each time point.

To further quantify the production of anti-ZIKV antibodies and to evaluate the evolution of the antibody response over time, an ELISA assay was developed using recombinant ZIKV NS1. A detectable NS1-specific antibody response was observed as early as 14 days post challenge (Fig 5a). Antibodies against ZIKV NS1 were also detected in pups originating from ZIKV challenged dams (Fig 5b). Anti-ZIKV NS1 antibody titers in fetal plasma were detected as early as 20 days post-infection (~40 days gestational age) and often exceeded the antibody titers of the infected dams by the end of gestation. These results suggest that while ZIKV challenge in pregnant guinea pigs did not result in detectable viremia, a subclinical infection sufficient to elicit a robust antiviral antibody response did occur. Additionally, the observed accumulation of anti-ZIKV NS1 in fetal plasma was consistent with previous reports of the timing and kinetics of maternal to fetal transfer of IgG in both guinea pigs and humans [36–38].

## Discussion

Since the association between ZIKV and developmental abnormalities such as microcephaly was recognized, several animal models of ZIKV infection have been evaluated. *In utero* ZIKV transmission has been observed in both nonhuman primates and, under some circumstances, mice. Multiple studies in macaques have observed that ZIKV viremia is prolonged during pregnancy and that the virus is transmitted transplacentally as it is in humans [7, 10, 12]. Murine models of congenital ZIKV syndrome have been largely dependent on immunosuppressed animals, where the pathogenicity of ZIKV, particularly with respect to transplacental transmission, is accentuated [15, 17, 39].

Similarities between human and guinea pig fetal development and pregnancy make the rodent an appealing system for the development of a small animal model of congenital ZIKV syndrome [19, 34, 40]. ZIKV infection in nongravid guinea pigs does cause low-level acute viremia. Levels of viremia observed in the current study were consistent with a previous report [26]. However, while peak viremia in acutely infected primates can range from 1X10^4^ to 5X10^7^ copies/ml plasma, the highest level of viremia that has been detected in a guinea pig was 2X10^4^ copies/ml [9, 11, 12, 26]. We observed that ZIKV infection in pregnant guinea pigs was well controlled and no detectable viremia or fetal transmission was observed. These results are consistent with experimental infection of guinea pigs with other flaviviruses, where viruses are typically not pathogenic but nonetheless immunogenic [41, 42]

The failure of wild type ZIKV to robustly infect guinea pigs and cause congenital disease is likely attributable to host restriction by innate antiviral pathways. Wild-type mice also restrict ZIKV replication, but inhibition of normal interferon α/β signaling increases ZIKV pathogenicity and facilitates transplacental transmission in mice [14, 17]. ZIKV NS5 has been observed to induce the proteasomal degradation of the interferon regulated transcriptional transactivator STAT2 in human cells but not mouse cells, and *Stat2*-null mice are highly susceptible to ZIKV infection [15]. While we found that a guinea pig cell line (JH4) supported ZIKV replication, we have previously observed that this cell line has an unusually weak PKR response compared to other cell lines and may have other defects in normal innate immune signaling [43]. A mouse-adapted strain of ZIKV has been reported but not fully described [44]. Evaluating this or another mouse-adapted strain of ZIKV for *in utero* viral transmission in guinea pigs would be an avenue for future work to develop this model, as would serially passaging ZIKV in guinea pigs in an attempt to enhance virulence.

ZIKV infection was highly immunogenic in guinea pigs and ZIKV-specific antibodies were detected in pup plasma after maternal challenge. This observation underscores the potential value of the small animal model for preclinical vaccine development and studies of the immunologic response to ZIKV during pregnancy if a virus capable of causing robust infection in guinea pigs is found or developed.

## Materials and methods

### Virus and cells

Vero cells (ATCC CCL-81) were propagated on Minimum Essential Media (MEM) supplemented with 10% fetal bovine serum (FBS), sodium pyruvate, MEM Non-Essential Amino Acids, and penicillin-streptomycin (ThermoFisher). ZIKV H/PF/2013 (passage 4) was obtained from the European Virus Archive and passaged twice on Vero cells to generate a working stock of virus [27]. Viral stocks were prepared using methodology originally developed for Dengue virus [45]. Briefly, passage 5 virus was prepared following infection of Vero cells at a MOI of 0.005. Virus containing cell culture supernatants were collected at 72 and 96 h.p.i., FBS added to a final concentration of 25% v/v, and the samples were clarified by centrifugation at 2700 X *g* at 4°C. Virus-containing media was aliquoted, flash frozen, and stored at -80°C. Passage 6 virus was further purified using a sucrose cushion. After infecting Vero cells at a MOI of 0.01, virus-containing media was collected at 72 h.p.i. and clarified as described above. The clarified medium was ultracentrifuged over a 20% sucrose cushion for 3.5 h at 130,000 X *g* at 4°C. After decanting the supernatant, the pellet was dried, resuspended in Dulbecco's phosphate-buffered saline (dPBS, Gibco), and syringe filtered (0.45 μm, Millipore) before aliquoting, flash freezing, and storing at -80°C.

Viral stocks and cell culture supernatant from growth curve analyses were titered by plaque assay. Samples were serially diluted in complete MEM and used to infect confluent monolayers of Vero cells. After adsorbing for 2 h, the virus-containing media was aspirated and the cells washed once with dPBS. The infected cells were overlaid with complete media containing 0.5% SeaPlaque agarose (Lonza). At 4 days post-infection, the cells were fixed with neutral buffered formalin, the agarose overlay was removed, and plates were stained with a crystal violet solution.

### Ethics statement

All animal procedures were conducted in accordance with protocols approved by the Institutional Animal Care and Use Committee (IACUC) at the University of Minnesota, Minneapolis (Protocol ID: 1604-33666A). With the oversight and approval of the IACUC, experimental protocols and endpoints were developed in strict accordance to the National Institutes of Health Office of Laboratory Animal Welfare (Animal Welfare Assurance #A3456-01), Public Health Service Policy on Humane Care and Use of Laboratory Animals, and United States Department of Agriculture Animal Welfare Act guidelines and regulations. Outbred Hartley guinea pigs were purchased from Elm Hill Laboratories (Chelmsford, MA). Guinea pigs were housed in Biosafety level 1 and 2 (BSL1 and 2) space that was maintained by the University of Minnesota Research Animal Resources, which is accredited through the Association for Assessment and Accreditation of Laboratory Animal Care, International (AAALAC). All procedures were conducted by trained personnel under the supervision of veterinary staff.

### Animal pathogenicity studies

Upon receipt, guinea pigs were assessed for guinea pig cytomegalovirus (GPCMV) serostatus by ELISA as previously described or using a commercial Guinea Pig CMV ELISA Kit (XpressBio IM-160C) [46]. Only GPCMV seronegative animals were used for ZIKV challenges. All guinea pigs were approximately 6 months of age at the time of ZIKV challenge. For timed mated animals, guinea pigs were initially bred at 3 months of age and mated during post-gestational estrus. This established the date of the first litter’s delivery as GA 0 of the second pregnancy. Pregnancy was confirmed by progesterone ELISA (DRG International); only animals with plasma progesterone concentrations exceeding 15 ng/ml at GA 20 were included in this study [47]. Animals were transferred from the breeding colony to BSL2 housing the day before viral challenge. Guinea pigs were injected subcutaneously in the dorsal neck with 0.5 ml of dPBS that contained 1X10^7^ or 1X10^8^ pfu of P6 ZIKV H/PF/2013. Control animals were sham injected with dPBS.

For non-pregnant animals, blood and plasma samples were collected every other day and the animals were euthanized at 14 days post infection. Brain and spleen samples were collected for viral load determination by qRT-PCR. Pregnant dams were challenged in two different experiments. In one experiment, animals were allowed to deliver and blood and plasma samples were collected weekly until the day of delivery, at which point the dams and pups were euthanized. In the second experiment, the dams were bled at 3, 10, 20, 30, and 40 days post infection and euthanized at 10, 20, 30, or 40 days post-challenge. The maternal and pup lungs, livers, spleens, and brains were collected for viral load determination and histologic analysis. Placenta and amniotic fluid were collected when possible. RNAlater (ThermoFisher) or RNAprotect animal blood tubes (Qiagen) was used to stabilize RNA in tissue and whole blood samples. All samples were stored at -80°C either immediately after collection or after an overnight stabilization at 4°C with RNAlater.

### Quantification of ZIKV viral loads and NS1-antigenemia

RNA was extracted from whole blood, plasma, and tissue using RNeasy Protect Animal Blood, QIAamp Viral RNA Mini, and RNeasy Mini kits (Qiagen). Tissue samples were homogenized using a FastPrep^®^-24 homogenizer and Lysing Matrix D (MP Biomedicals). qPCR primers and probes ZIKV_1086, ZIKV_1162c, ZIKV_1107_P were synthesized by Integrated DNA Technologies [48]. Viral loads were quantified by qRT-PCR using the SuperScript^™^ III Platinum^™^ One-Step qRT-PCR Kit (Thermo Fisher) and a LightCycler 480 (Roche) instrument as previously described [10]. To determine the concentration of ZIKV RNA in a sample, a synthetic DNA fragment corresponding to the H/PF/2013 genome from positions 1086 to 1107 was amplified and subcloned into pCR4-TOPO (Thermo Fisher). RNA standards were synthesized using a MAXIscript^™^ T7/T3 Transcription Kit (Thermo Fisher), quantified by absorbance and Quant-iT Ribogreen RNA Assay Kit (Life Technologies), and serially diluted to generate in-run standard curves for qRT-PCR analysis. To quantify ZIKV NS1-antigenemia, plasma from guinea pigs was subjected to a Zika Virus NS1 ELISA (BioFront Technologies).

### Immunoblots

Samples of 0.5 μg of purified, recombinant ZIKV NS1 or Env (Meridian Life Science R01635, R01636) or 5 μg of ZIKV H/PF/2013 viral particles purified over a sucrose cushion were denatured, resolved on Novex 12% Tris-Glycine Mini Gels (ThermoFisher), and transferred to an Immun-Blot PVDF Membrane (BioRad #1620177). After rinsing twice with PBS containing 0.05% Tween-20, the membranes were incubated overnight in a 2% solution of Amersham ECL Prime Blocking Reagent (GE Healthcare Life Sciences) at 4°C. The membranes were washed three times with PBS-Tween and incubated for 2 hours at room temperature with guinea pig plasma samples that had been diluted 1:500 in 2% blocking reagent. Plasma samples had been collected from pregnant guinea pigs either the day before ZIKV challenge or at the day of delivery. The membranes were washed three times with PBS-Tween prior to a 1 hour room temperature incubation with secondary rabbit anti-guinea pig IgG-peroxidase (Sigma A5545) diluted 1:5000 in 2% blocking reagent. After three final washes with PBS-Tween, the immunoblots were incubated with SuperSignal West Dura Extended Duration Substrate (ThermoFisher 34075), exposed to film, and developed.

### α-ZIKV NS1 ELISA

The guinea pig IgG response to ZIKV NS1 was measured by ELISA. Recombinant ZIKV NS1 (Meridian Life Science R01636) was diluted in PBS containing 0.05% sodium azide and used to coat 96 well plates; 133 ng of recombinant protein was used to coat each well. After incubating overnight at room temperature, unbound protein was aspirated and the plates were washed with buffer containing 0.5 M NaCl, 13.3 mM Na_2_HPO_4_, 3 mM KH_2_PO_4_, and 0.05% Tween-20. The coated plates were blocked for 2 hours with blocking buffer (2% skim milk diluted in washing buffer). The blocking buffer was discarded, the plates washed three times, and plasma serially diluted in blocking buffer was added to the plates. After incubating the samples for 2 hours at room temperature, the diluted plasma was aspirated and the plates washed 3 times. Rabbit anti-guinea pig IgG-peroxidase (Sigma A5545) was diluted 1:5000 in blocking buffer, added to the plates, and incubated for 1 hour at room temperature. After aspirating the secondary antibody and washing the plates 6 times, TMB Chromogen Solution (Thermo Fisher) was added to develop the assay for 15 minutes at room temperature. A 10% solution of sulfuric acid was added to stop the peroxidase reaction and the absorbance at 450nm was read. Antibody titers were defined as the maximum dilution of plasma to produce an absorbance at 450nm above background.
